# Genetic Variability and Evolutionary Dynamics of A(H1N1)pdm09 in Italy Before, During, and After the COVID-19 Pandemic

**DOI:** 10.3390/pathogens15090920

**Published:** 2026-09-01

**Authors:** Maria Perra, Ilaria Deplano, Ilenia Azzena, Chiara Locci, Giancarlo Ceccarelli, Dong Keon Yon, Francesco Branda, Massimo Ciccozzi, Marco Casu, Alessandra Borsetti, Daria Sanna, Fabio Scarpa

**Affiliations:** 1 Department of Biomedical Sciences, University of Sassari, 07100 Sassari, Italy; m.perra9@phd.uniss.it (M.P.); i.deplano@phd.uniss.it (I.D.); darsanna@uniss.it (D.S.); 2 Department of Veterinary Medicine, University of Sassari, 07100 Sassari, Italy; iazzena@uniss.it (I.A.); c.locci3@phd.uniss.it (C.L.); 3 Department of Public Health and Infectious Diseases, University of Rome Sapienza, 00185 Rome, Italy; giancarlo.ceccarelli@uniroma1.it; 4 Genomics, AI, Bioinformatics, Infectious Diseases, Epidemiology Group (GABIE), 00128 Rome, Italy; f.branda@unicampus.it (F.B.); m.ciccozzi@unicampus.it (M.C.); 5 Migrant and Global Health Research Organization (Mi-HeRO), 00185 Rome, Italy; 6 Azienda Ospedaliero Universitaria Umberto I, 00161 Rome, Italy; 7 Center for Digital Health, Medical Science Research Institute, Kyung Hee University College of Medicine, Seoul 05278, Republic of Korea; yonkkang@gmail.com; 8 Department of Pediatrics, Kyung Hee University Medical Center, Kyung Hee University College of Medicine, Seoul 05278, Republic of Korea; 9 Unit of Medical Statistics and Molecular Epidemiology, University Campus Bio-Medico of Rome, 00128 Rome, Italy; 10 Department of Innovation, University of Sassari, 07026 Olbia, Italy; marcasu@uniss.it; 11 National HIV/AIDS Research Center (CNAIDS), Istituto Superiore di Sanità, 00161 Rome, Italy

**Keywords:** A(H1N1)pdm09, influenza A virus, genetic variability, phylodynamics, genomic surveillance, hemagglutinin, neuraminidase, COVID-19 pandemic, viral evolution, Italy

## Abstract

Influenza A(H1N1)pdm09 remains one of the predominant seasonal influenza viruses worldwide and continues to evolve under the combined effects of host immunity, vaccination, and changing epidemiological conditions. However, the long-term impact of the COVID-19 pandemic on its evolutionary dynamics remains poorly understood. We investigated the genetic variability and phylodynamic evolution of the hemagglutinin (HA) and neuraminidase (NA) genes of Italian A(H1N1)pdm09 viruses collected between 2009 and 2026. Time-calibrated phylodynamic analyses, Bayesian Skyline Plots (BSPs), Lineages Through Time (LTT) graphs, principal component analysis (PCA), and codon-based selection analyses were used to characterize long-term evolutionary patterns. Both HA and NA followed continuous evolutionary trajectories, with a marked post-2020 genetic shift associated with the emergence of 6B.1A.5a.2-related clades. HA showed greater evolutionary variability than NA, whereas selection analyses identified only one positively selected site in HA and none in NA, consistent with reduced adaptive diversification in recent strains. Phylodynamic analyses revealed a marked decline in effective population size and lineage accumulation during the COVID-19 pandemic, followed by renewed expansion after 2022. Overall, Italian A(H1N1)pdm09 viruses exhibited reduced genetic diversity, limited evidence of positive selection, and coordinated genomic restructuring following the pandemic. These findings provide new insights into the long-term evolutionary dynamics of A(H1N1)pdm09 in Italy and reinforce the importance of sustained genomic surveillance for anticipating evolutionary changes and informing evidence-based public health strategies.

## 1. Introduction

Influenza A viruses, classified within the *Orthomyxoviridae* family, are segmented, single-stranded, negative-sense RNA pathogens capable of infecting a wide range of hosts, including wild avian species, non-human mammals, and humans [1]. Influenza A viruses are classified according to combinations of the surface glycoproteins hemagglutinin (HA) and neuraminidase (NA), which largely determine host tropism and antigenic properties [2]. Genetic changes at critical sites of these glycoproteins may arise through amino acid substitutions (antigenic drift) or through reassortment (antigenic shift) [3]. Antigenic drift and, less frequently, antigenic shift drive the continuous evolution of influenza A viruses and underlie seasonal epidemics as well as occasional pandemics [3,4]. The genome of influenza A viruses consists of eight segments, each contributing differently to viral fitness; among these, HA and NA are the major antigenic determinants and are therefore essential for interpreting the evolutionary dynamics of circulating viruses [5]. Several studies have shown that HA evolves more rapidly than NA, reflecting stronger selective pressure exerted by human immunity, whereas NA tends to maintain greater functional stability and contributes mainly to viral transmissibility and release efficiency [6]. For these reasons, the analysis of the HA and NA segments represents a particularly informative approach for investigating the genetic evolution and diversification of A(H1N1)pdm09 viruses, enabling the identification of mutations associated with viral adaptation, immune escape, and changes relevant to seasonal vaccine effectiveness [7].

The evolution of A(H1N1)pdm09 is primarily driven by the accumulation of mutations generated by the error-prone viral RNA-dependent RNA polymerase and by the selective pressures imposed by host immunity [8,9]. In circulating lineages, antigenic drift progressively modifies HA and NA, potentially reducing antibody recognition and affecting vaccine effectiveness and transmission dynamics [4,10]. Although less frequent than antigenic drift, reassortment remains an important component of influenza A virus evolution [11]. The 2009 pandemic strain itself originated through reassortment among swine, avian, and human influenza viruses, illustrating how segmented genomes can generate novel viral constellations with altered fitness and transmissibility [12,13]. Because swine and avian reservoirs continue to provide a broad pool of influenza genetic diversity, integrated surveillance of human and animal populations remains essential for detecting reassortant viruses with zoonotic or pandemic potential [14,15,16,17,18,19,20,21]. The COVID-19 pandemic further altered influenza circulation and vaccination behaviors, creating atypical epidemiological conditions that may have influenced the recent evolutionary trajectory of the virus. In Italy, the 2020–2021 influenza season was characterized by an unprecedented reduction in influenza virus circulation. National virological surveillance reported no influenza-positive samples among 6818 specimens tested between week 42 of 2020 and week 16 of 2021, compared with a mean influenza positivity rate of 28.8% during the corresponding period of the previous five seasons [22]. This near-complete interruption of influenza circulation was also reflected in influenza-like illness surveillance, which remained at interseasonal levels throughout the season [23]. During the same period, influenza vaccination coverage in Italy increased from 16.8% in 2019–2020 to 23.7% in 2020–2021, with a particularly marked increase among adults aged ≥65 years, from 54.6% to 65.3% [22]. However, this increase in vaccination coverage alone is unlikely to explain the almost complete disappearance of influenza circulation, which was more plausibly associated with the combined effects of non-pharmaceutical interventions, reduced population mobility and social contact, and other pandemic-related changes in influenza transmission dynamics [24,25]. Advances in next-generation sequencing and whole-genome analyses have substantially improved the resolution of influenza genomic surveillance, enabling the identification of emerging variants and the reconstruction of transmission and evolutionary patterns [26]. The integration of genetic, structural, clinical, and epidemiological information is increasingly important for interpreting how specific substitutions affect viral adaptation, immune escape, and disease severity [27,28,29]. In addition to antigenic drift and shift, the evolution of the A(H1N1)pdm09 virus has also been influenced by seasonal vaccination [30,31]. Since its emergence in 2009, the virus has been included annually in influenza vaccines, contributing to selective pressure mainly on the HA protein and, to a lesser extent, on NA [4,32,33]. Mass vaccination may have favored the persistence of antigenically adapted lineages and the periodic replacement of circulating strains [34]. The COVID-19 pandemic further altered influenza circulation and vaccination behaviors, creating atypical epidemiological conditions that may have influenced the recent evolutionary trajectory of the virus. In this context, genomic surveillance remains essential for identifying emerging variants and supporting the annual update of seasonal vaccines and pandemic-risk assessment [35].

The evolutionary impact of these processes extends beyond seasonal circulation: under specific ecological and immunological conditions, they have led to pandemic events, as demonstrated by the four influenza pandemics of the last century (1918, 1957, 1968 and 2009) [36,37,38,39,40].

The A(H1N1)pdm09 virus emerged in 2009 following reassortment among North American and Eurasian swine influenza viruses, generating a novel genetic constellation previously undetected in humans [3]. After its emergence in Mexico, sustained human-to-human transmission rapidly resulted in the first influenza pandemic of the twenty-first century, prompting the World Health Organization to declare a phase 6 pandemic and triggering an immediate international public health response [8,41,42,43,44].

In Italy, A(H1N1)pdm09 rapidly became one of the predominant circulating influenza A viruses after the 2009 pandemic and has continued to circulate seasonally, contributing substantially to influenza-associated morbidity and mortality [45]. During the 2024–2025 season in Italy, 601 severe laboratory-confirmed influenza cases requiring intensive care admission were reported, of which 134 resulted in death. The A(H1N1)pdm09 subtype was most strongly associated with severe disease and deaths among patients with confirmed influenza [46].

Several molecular epidemiology studies have investigated the evolutionary dynamics of the A(H1N1)pdm09 virus in the years following its emergence, providing important insights into its post-pandemic diversification [3,8,47], underscoring the importance of sustained genomic surveillance to detect emerging variants with potential public health relevance.

However, few studies have examined how long-term vaccination, the COVID-19 pandemic, and post-pandemic immunological shifts have jointly shaped the recent evolutionary dynamics of A(H1N1)pdm09. Given the pandemic potential of this virus, continuous genetic monitoring is essential to ensure robust surveillance capable of identifying changes in viral fitness that may pose a pandemic threat and to anticipate the emergence of circulating strains relevant to public health. Integrating genomic monitoring with vaccination data, such as coverage, vaccine match, and immune landscape, will be increasingly important for interpreting evolutionary trends in the post-pandemic era.

In this context, the present study aimed to perform a genetic and phylodynamic investigation of the HA and NA sequences of A(H1N1)pdm09 Italian isolates to determine the evolutionary trend of the human pandemic influenza virus circulating in Italy from 2009 to 2026. Specifically, the research sought to characterize viral evolution across different epidemiological phases between 2009 and 2026, including the period of reduced influenza circulation associated with COVID-19, and to assess whether the evolutionary patterns observed align with the major ecological and immunological shifts occurring over these years.

## 2. Materials and Methods

### 2.1. Dataset Assembly and Alignment

All sequence data used in this study were retrieved from the GISAID database [48]. We included only human influenza A(H1N1)pdm09 sequences originating from Italy. To capture the full evolutionary history of this lineage, we selected sequences sampled between 2009—its year of emergence—and 2026. Only records available as open data (i.e., without embargo restrictions) and accompanied by complete metadata were retained. To ensure high data quality, we further restricted the dataset to high-coverage sequences with a minimum of 90% genome length. Four independent datasets were assembled according to the analytical objectives (for details, see Supplementary File S1). The first datasets include all Italian HA and NA sequences collected between 2009 and 2026 (HA, n = 1315, https://doi.org/10.55876/gis8.260805hc; NA, n = 1107, https://doi.org/10.55876/gis8.260805xg). This comprehensive dataset was used for the time-calibrated phylodynamic reconstruction, the Bayesian Skyline Plot (BSP) and Lineages Through Time (LTT) analyses and the assessment of genetic diversity metrics.

A second employed dataset includes all HA and NA sequences sampled from October 2025 to February 2026 (HA, n = 151, https://doi.org/10.55876/gis8.260805as; NA, n = 160, https://doi.org/10.55876/gis8.260805qz). This recent dataset was specifically used for selection analyses aimed at detecting codon sites under selective pressure.

All datasets were aligned separately using MAFFT v7.471 with the L-INS-I algorithm [49,50], followed by manual inspection and refinement in Unipro UGENE v.53.1 [51]. The best fitting nucleotide substitution model for each dataset was identified with jModelTest v2.1.1 using a maximum likelihood-based model selection procedure [52]. Phylogenetic signal was evaluated by likelihood mapping using Tree-Puzzle v.5.3, with 10,000 randomly generated quartets analyzed separately for each dataset [53].

### 2.2. Genetic Structure

Genetic structure was evaluated through principal component analysis (PCA) conducted in R on the full dataset (2009–2026). The same p distance matrices were transformed into principal components using the adegenet package [54]. This approach provided a multivariate representation of sequence distribution, enabling the identification of potential clusters or genetically distinct groups. After computing the PCA, we plotted the PC1 values as a time ordered scatterplot and applied LOESS smoothing in R to obtain continuous evolutionary trajectories. This visualization enabled a clearer interpretation of clade specific dynamics and temporal shifts in genetic structure.

### 2.3. Phylodynamic Simulation

For the full datasets (2009–2026), the phylodynamic reconstruction was performed in BEAST v1.10.4 [55]. Independent Markov chain Monte Carlo (MCMC) runs of 400 million generations were conducted under multiple combinations of molecular clocks and demographic models. Model comparison was carried out through Bayes Factor evaluation based on marginal likelihood estimates computed in Tracer v1.7.2 [56].

The Bayesian Skyline Plot (BSP) was used to infer temporal changes in effective population size, whereas Lineages Through Time (LTT) plots were generated to visualize fluctuations in lineage accumulation through time and to identify periods of accelerated or reduced diversification. BEAST software was employed to perform both the BSP and the LTT analyses, while Tracer v1.7.2 was used to generate and visualize the corresponding plots. Together, these complementary approaches provided a broad overview of long-term demographic trends and allowed the detection of shifts in the evolutionary dynamics of the viral population across the study period.

### 2.4. Genetic Diversity

Genetic diversity of the HA and NA segments was estimated in R using the ape [57] and pegas [58] packages, calculating for each epidemiological period (pre-COVID-19, COVID-19, and post-COVID-19) the number of conserved and polymorphic sites, the percentage of polymorphic sites, singleton and parsimony-informative sites, non-ACGT positions, the number of haplotypes, haplotype diversity, nucleotide diversity (π), and p-distance. These parameters were used to assess whether temporal changes in genetic diversity reflected fluctuations in viral population size.

### 2.5. Selective Pressure

For the recent dataset (October 2025–2026), selective pressures acting on codon sites were investigated using FUBAR (Fast, Unconstrained Bayesian AppRoximation) [59] implemented on the Datamonkey platform [60]. Analyses were performed separately for HA and NA to identify sites evolving under pervasive positive or negative selection and to characterize the most recent evolutionary patterns of the virus.

## 3. Results

**Figure 1:**
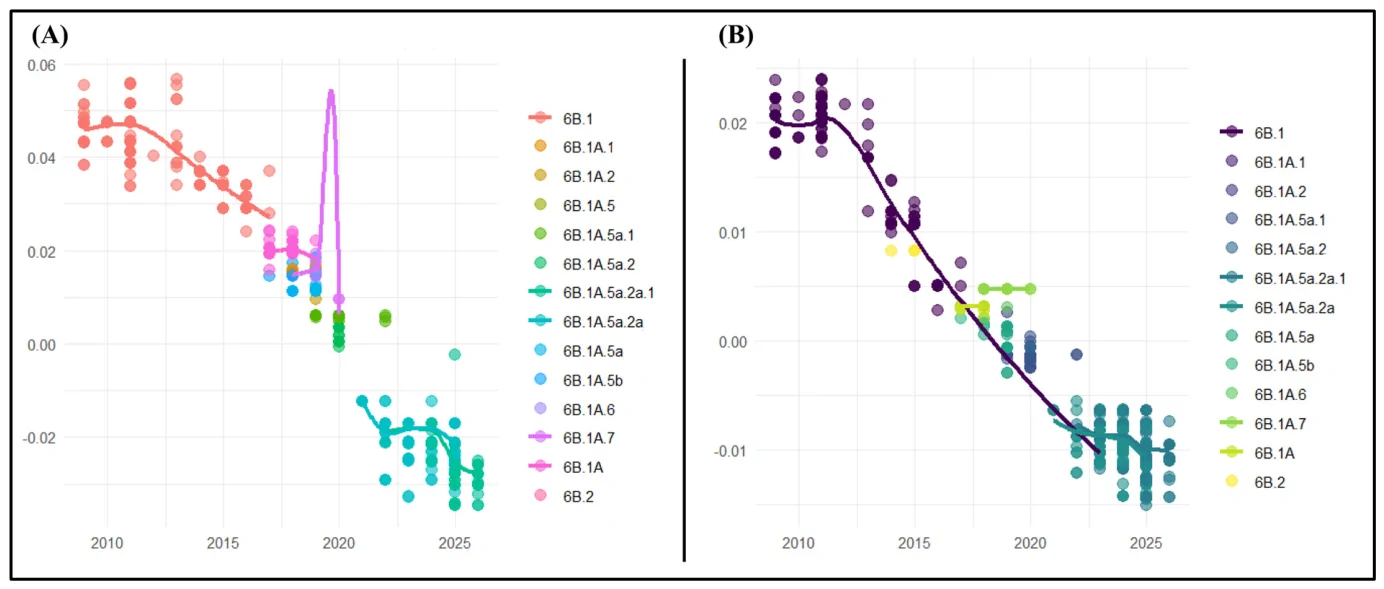
Temporal PCA trajectories of Italian A(H1N1)pdm09 viruses. LOESS-smoothed PC1 values are shown for (**A**) HA and (**B**) NA sequences. The *x-axis* represents sampling year, and the *y-axis* represents genetic distance (PC1). Points are colored by clade designation.

The principal component analysis (PCA) of the HA and NA segments revealed clear evolutionary trajectories of Italian A(H1N1)pdm09 viruses between 2009 and 2026 (Figure 1). In both genes, the first principal component (PC1) captured a gradual temporal shift, consistent with continuous genetic drift rather than discrete clustering.

For the HA segment, early-pandemic and post-pandemic strains belonging to clade 6B.1 occupied higher PC1 values during 2009–2015, followed by increased dispersion after 2017 associated with the emergence of multiple 6B.1A-derived subclades. HA trajectories showed transient divergence of specific subclades, particularly around the appearance of 6B.1A.7. In contrast, the NA segment displayed a more linear and compact temporal trajectory, with lower dispersion and a more constrained evolutionary pattern. Despite these differences, both segments showed a pronounced post-2020 displacement in the PCA space. Recent strains, predominantly associated with 6B.1A.5a.2-related clades, formed distinct clusters separated from earlier lineages, indicating substantial genetic turnover following the COVID-19 period. Together, the concordant shifts observed in HA and NA indicate coordinated temporal changes across both segments.

**Figure 2:**
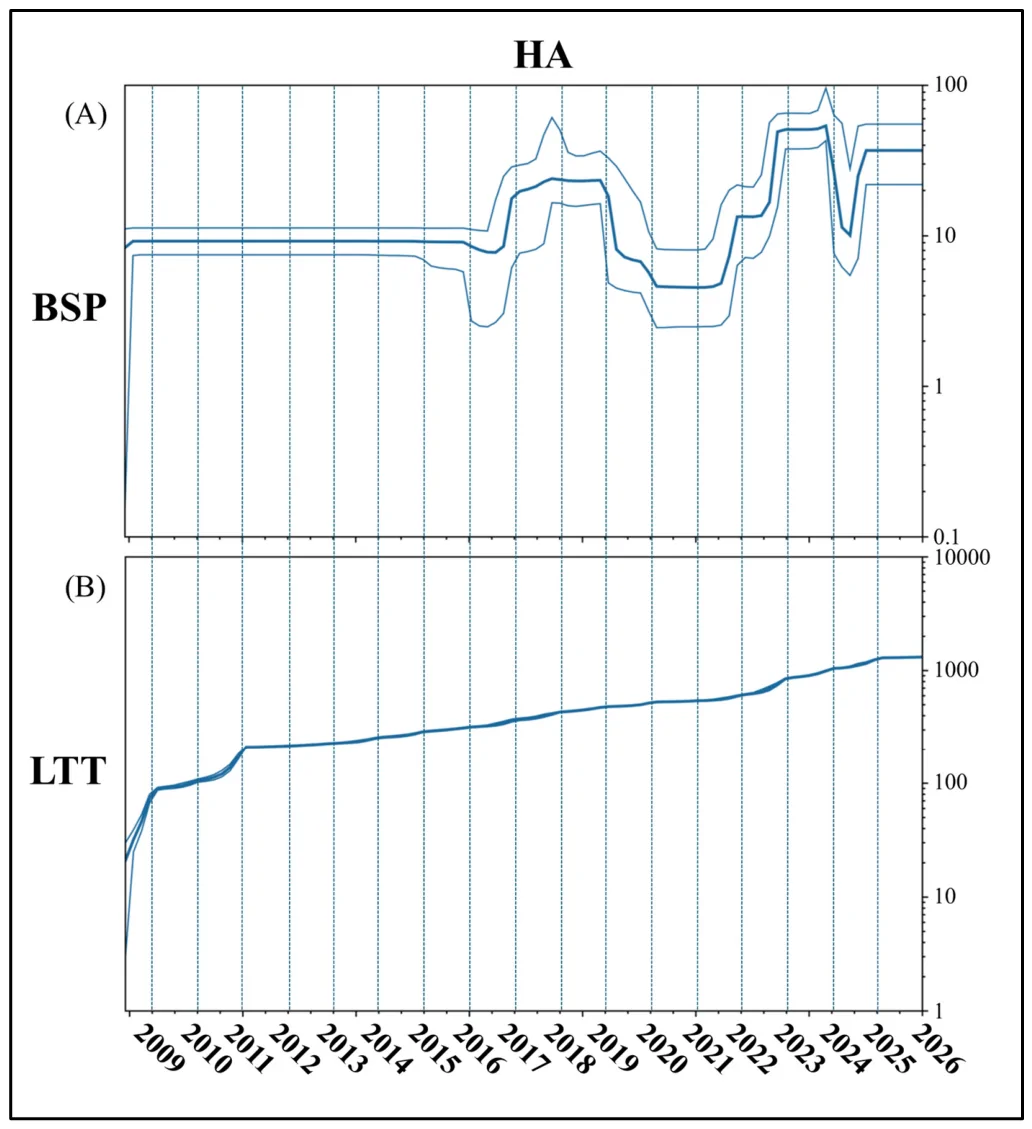
Bayesian Skyline Plot and Lineages Through Time of the HA gene. The viral effective population size (**A**) and the number of lineages (**B**) on the *y-axes* are shown as functions of years (*x-axes*). In both panels, the central solid line represents the median estimate, while the upper and lower lines indicate the corresponding 95% Highest Posterior Density (HPD) interval.

**Figure 3:**
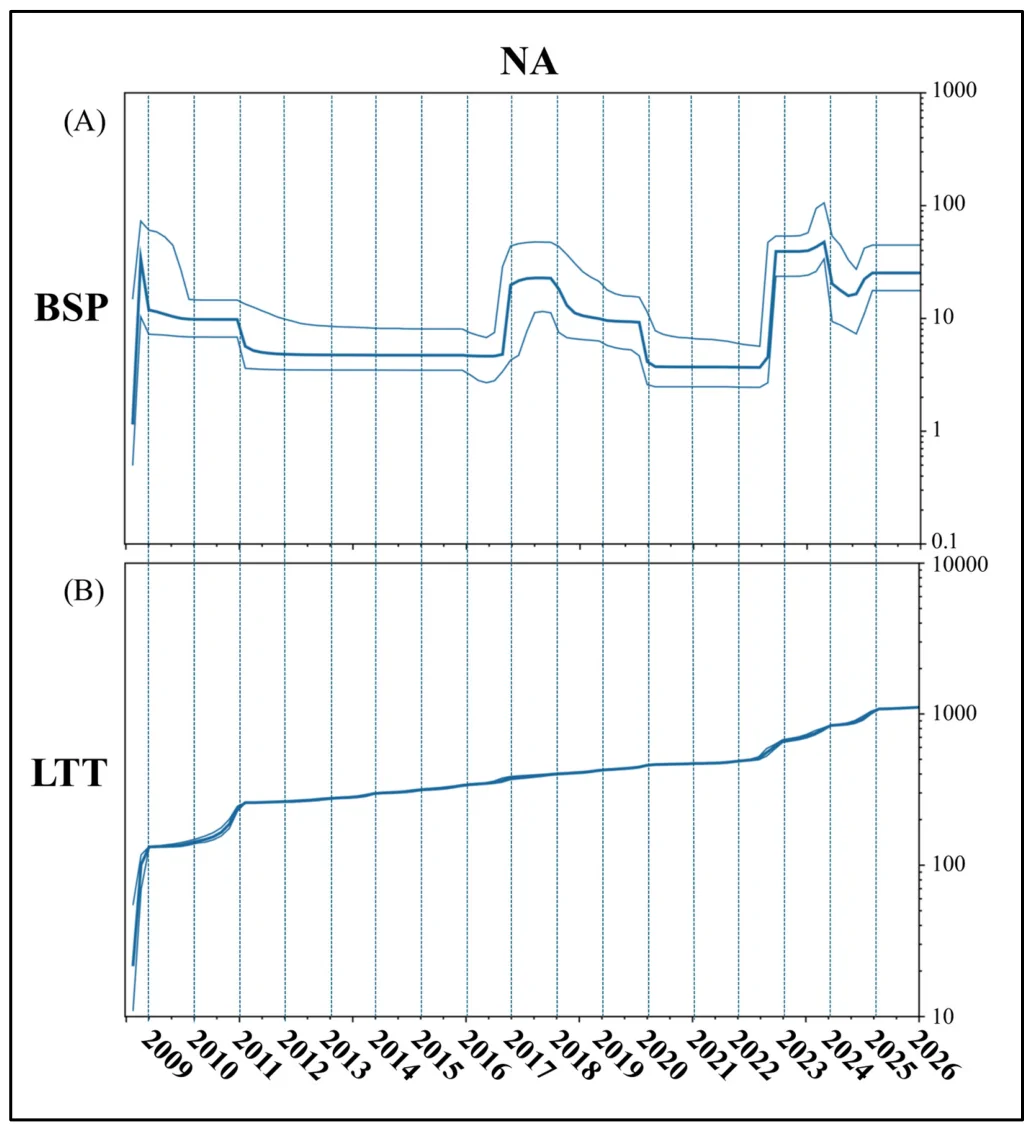
Bayesian Skyline Plot and Lineages Through Time of the NA gene. The viral effective population size (**A**) and the number of lineages (**B**) on the *y-axes* are shown as functions of years (*x-axes*). In both panels, the central solid line represents the median estimate, while the upper and lower lines indicate the corresponding 95% Highest Posterior Density (HPD) interval.

The Bayesian Skyline Plot (BSP) analyses revealed distinct temporal demographic patterns for the HA (Figure 2A) and NA (Figure 3A) genomic segments. In the HA dataset, the effective population size remained relatively stable between 2009 and 2016, followed by a marked increase around 2017–2018. A sharp decline occurred between 2020 and early 2021. However, the curve had already begun to rise again in the second half of 2021, with a more pronounced increase from 2022 onwards, ultimately reaching the highest values in the most recent years.

The NA BSP (Figure 3A) showed a more stable demographic profile. After an initial adjustment phase following 2009, the effective population size remained largely constant, with moderate increases around 2017–2018 and again after 2022. A reduction in effective population size was also detected between 2020 and 2021, but the magnitude of demographic fluctuations in NA was considerably lower than that observed for HA.

The Lineages Through Time (LTT) (Figure 2B) graph was consistent with the BSP results. Both HA and NA exhibited rapid lineage accumulation during the early years of the dataset (2009–2011), followed by a more gradual increase over time (Figure 2B and Figure 3B). A reduction in the slope of the curves was observed between 2020 and 2021, indicating slower lineage accumulation during this interval. From the second half of 2021, and more clearly from 2022 onwards, both segments showed renewed increases in lineage numbers, with HA displaying a steeper rise than NA. Overall, HA exhibited more dynamic and variable lineage accumulation patterns compared with the more stable trajectory observed for NA. For details on the genetic variability of the genes HA and NA see Table 1. Overall, both genes showed a marked reduction in genetic diversity during the pandemic period, followed by divergent post-pandemic trajectories. HA exhibited a partial recovery of nucleotide diversity, although values remained below pre-pandemic levels, whereas NA showed a further decline in nucleotide diversity despite the increase in polymorphic sites and haplotype number.

**Figure 4:**
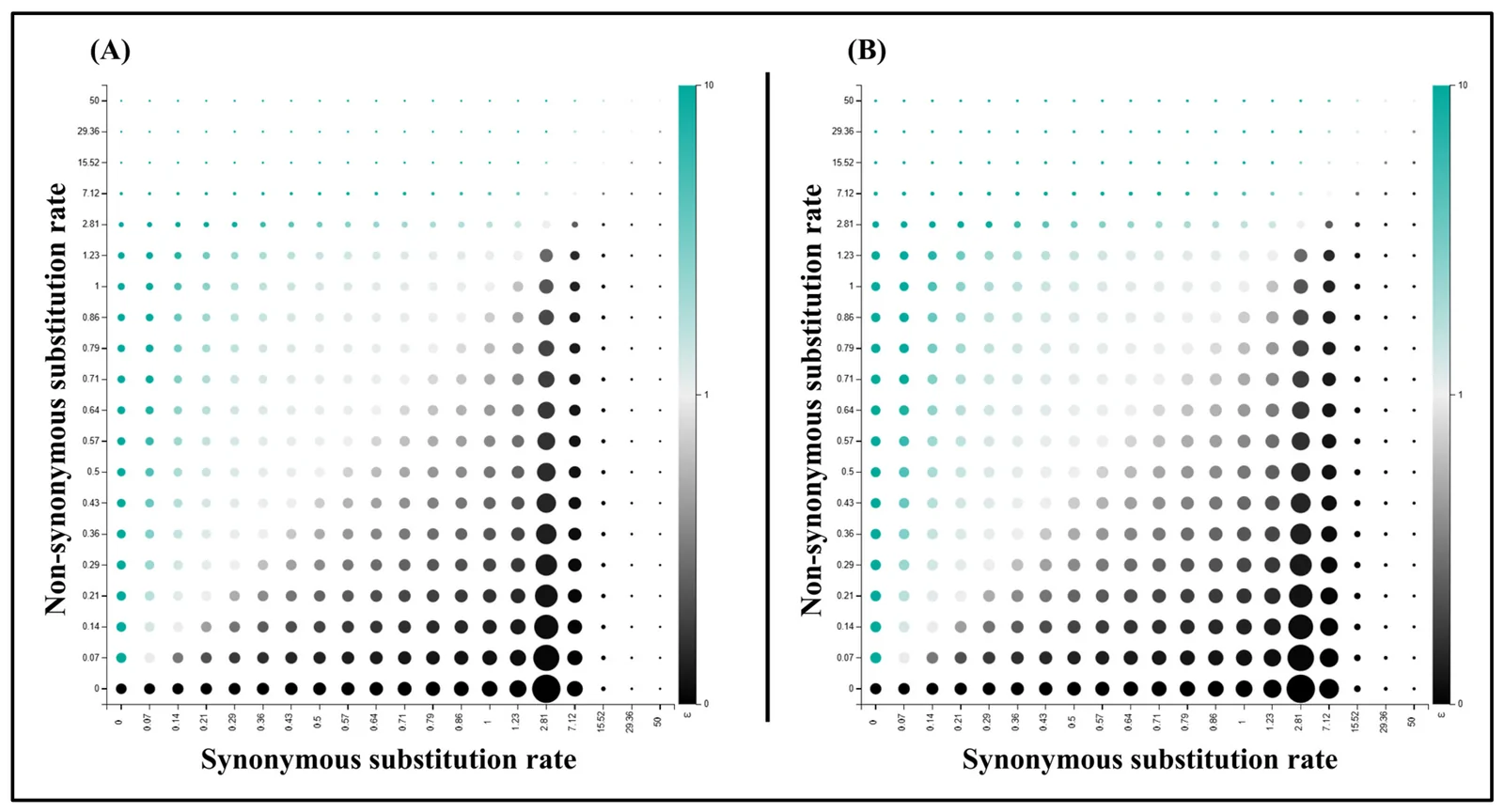
Posterior rate distribution across a discretized rate grid of the ω parameter for (**A**) HA 2025-26 and (**B**) NA 2025-26. The colored bar on the right of each graph indicates the strength of selection. ω > 1 represents positive selection, ω = 1 represents neutrality, and ω < 1 represents negative selection. The *x-axes* indicate synonymous substitution rate, and the *y-axes* indicate non-synonymous substitution rate. The size of each dot corresponds to the posterior weight assigned to each grid point, while the color indicates the strength of selection. Sites under positive selection are highlighted in green, and those under negative selection are highlighted in black. See Table 2 for details.

The analysis of selective pressure (Figure 4, Table 2) identified one positively selected site in the HA alignment. This site, located at amino acid position 468 (nt 1402–1404), showed a markedly higher non-synonymous substitution rate compared with the synonymous rate (β = 5.498 vs. α = 0.793), resulting in a β–α difference = 4.705. The posterior probability of positive selection at this site was high (Prob [α < β] = 0.907), supported by a Bayes Factor = 17.032, which exceeds the threshold for significance. No other HA sites met the criteria for positive selection. No additional HA sites showed evidence of positive selection. Conversely, the analysis detected 26 sites under purifying selection in HA and 10 in NA, indicating that the evolutionary dynamics of both genes are largely dominated by strong functional constraints (Table 2).

## 4. Discussion

Genetic variability represents a key indicator of the evolutionary processes that shape the circulation and adaptation of influenza viruses. Understanding how these changes accumulate over time is essential for interpreting transmission dynamics, immune escape, and the impact of selective pressures exerted by the host population. In this context, our study analyzed the genetic evolution of the A(H1N1)pdm09 virus across its entire period of human circulation (2009–2026), with particular attention to the phase influenced by the COVID-19 pandemic. The aim was to assess whether containment measures, behavioral changes, and shifts in population immunity associated with the pandemic left detectable signatures in the genetic variability of the influenza virus.

Against this background, our analyses provide a long-term view of the genetic and phylodynamic changes affecting the HA and NA genes of A(H1N1)pdm09 viruses circulating in Italy between 2009 and 2026. Comparative analyses of the HA and NA segments revealed both shared and segment-specific evolutionary patterns in Italian A(H1N1)pdm09 viruses circulating between 2009 and 2026. Principal component analysis (PCA) showed that both genes followed a continuous evolutionary trajectory rather than forming discrete temporal or geographic clusters, consistent with sustained viral circulation and extensive mixing within the Italian population. Despite this overall similarity, HA exhibited markedly greater dispersion and a more complex trajectory than NA, reflecting the stronger antigenic selection pressures acting on hemagglutinin, the primary target of host immunity. In contrast, NA followed a more compact and canalized evolutionary path, in line with its more conserved functional constraints and its compensatory role in maintaining viral fitness.

A key feature shared by both segments was the clear post-2020 displacement toward a distinct genetic space dominated by 6B.1A.5a.2-related clades. The parallel movement of HA and NA toward the same post-COVID-19 cluster indicates that this transition was not restricted to antigenic changes in HA alone but involved a broader genomic turnover. Such coordinated displacement across independent segments suggests that the evolutionary dynamics of influenza may have been influenced by the epidemiological conditions associated with the COVID-19 pandemic, including reduced viral circulation, population-level bottlenecks, and subsequent lineage replacement following the relaxation of non-pharmaceutical interventions. The transient elevation observed in the HA trajectory during the pre-pandemic years, particularly around the emergence of clades such as 6B.1A.7, was far less pronounced in NA, underscoring the differential selective pressures acting on the two surface glycoproteins and highlighting the importance of multi segment analyses for capturing the full complexity of influenza evolution.

Selection analyses further supported this interpretation. FUBAR identified only a single positively selected site in HA (site 468) and none in NA during the 2025–2026 season. This represents a striking reduction in adaptive signals compared with earlier years: Scarpa et al. [3] reported two positively selected HA sites in 2009 (positions 41 and 177) and three in 2023 (sites 22, 123, and 513), as well as three positively selected NA sites in 2009 (positions 248, 286, and 455) and one in 2023 (site 339). The limited number of positively selected sites detected in the current dataset may indicate reduced detectable adaptive diversification, although this finding should be interpreted cautiously given the sensitivity and temporal resolution of site-based selection analyses. The complete lack of positively selected sites in NA further supports the predominance of purifying selection acting on this gene and is consistent with the comparatively conserved evolutionary trajectory observed for NA.

These findings are compatible with an evolutionary framework potentially influenced by long-term vaccination strategies. The continuous inclusion of A(H1N1)pdm09 in seasonal influenza vaccines may have contributed to maintaining relatively stable immune pressure on the HA protein, promoting the selection of variants capable of partially escaping pre-existing immunity [4,33]. However, the near absence of positively selected sites in the 2025–2026 dataset suggests a phase of relative antigenic stability, likely driven by the predominance of a single lineage (6B.1A.5a.2a.1) and by a high degree of immunological homogeneity in the population. This scenario may also reflect an improved match between vaccine strains and circulating viruses, resulting in reduced selective pressure for the emergence of escape variants [61]. At the same time, the renewed increase in genetic diversity observed after 2021 underscores the importance of continued surveillance and vaccination programs. Integrating evolutionary data with vaccination strategies will therefore be essential to improve strain selection predictability and to support the development of more broadly protective influenza vaccines [62]. This evolutionary stabilization is also reflected in the demographic patterns inferred from phylodynamic analyses. Indeed, results highlighted substantial differences in the evolutionary dynamics of the HA and NA genomic segments. The stronger fluctuations observed in the HA Bayesian Skyline Plot suggest that this segment experienced repeated phases of expansion and contraction in effective population size, consistent with a highly dynamic evolutionary process. A notable finding was the marked reduction in effective population size observed between 2020 and 2021 in both HA and NA datasets, coinciding with the global COVID-19 pandemic. This contraction likely reflects the dramatic decrease in influenza virus circulation reported worldwide during the implementation of non-pharmaceutical interventions, including travel restrictions, social distancing, mask use, and school closures. These measures significantly reduced the transmission of respiratory viruses, producing a temporary bottleneck in viral genetic diversity and lineage persistence. This pattern is consistent with recent studies [63,64], which documented a pronounced COVID-19-associated bottleneck in influenza viruses and the near-elimination of several lineages during this period. Both genomic segments showed evidence of renewed expansion beginning in 2022–2023, consistent with the global resurgence of influenza circulation after the relaxation of public health restrictions. However, the post-pandemic recovery appeared more rapid and extensive in HA, further supporting the hypothesis that HA undergoes faster diversification and lineage turnover relative to NA. The LTT analyses were consistent with these observations. Although lineage accumulation continued throughout the study period, the reduced slope observed during the pandemic years suggested a slowdown in the generation and maintenance of viral diversity. The subsequent acceleration in lineage accumulation after 2022 indicates a re-establishment of viral circulation and diversification dynamics. Beyond the phylodynamic evidence, additional support for these trends comes from the analysis of genetic diversity in the HA and NA segments. Before the pandemic, both genes displayed high levels of polymorphism, haplotype richness, and nucleotide diversity, consistent with the substantial genetic variability observed in our dataset. During the COVID-19 period, all diversity metrics showed a marked reduction, reflecting a strong demographic bottleneck associated with the profound decline in influenza circulation during the implementation of non-pharmaceutical interventions [65]. In the post-pandemic period, genetic diversity increased again, with a substantial recovery in the number of haplotypes and polymorphic sites; however, nucleotide diversity—particularly in NA—remained lower than pre-pandemic levels. The divergent post-pandemic trajectories of nucleotide diversity in HA and NA deserve specific consideration. While HA showed a partial recovery of nucleotide diversity after the pandemic, NA exhibited a further decrease despite the concomitant increase in the number of polymorphic sites and haplotypes. This indicates that the recovery of genetic richness observed in NA did not correspond to an increase in average pairwise sequence divergence. Such a pattern is consistent with the more compact evolutionary trajectory observed for NA in the PCA and with its comparatively stable phylodynamic profile, supporting the different post-pandemic evolutionary dynamics of the two segments. Nevertheless, differences in sequence availability and sampling intensity among periods may also have influenced diversity estimates; therefore, the contrasting post-pandemic patterns of HA and NA should not be interpreted exclusively as reflecting biological differences between the two segments.

More broadly, the post-pandemic recovery in genetic diversity mirrors the resurgence of viral circulation and the demographic expansion highlighted by the BSP and LTT analyses, suggesting that the virus resumed diversification after the pandemic, although this recovery did not correspond to a complete restoration of pre-pandemic levels of genetic diversity. Consistently, the limited number of positively selected sites detected in the current dataset provides no evidence of strong ongoing adaptive diversification at the codon level. Together, these findings support the view that HA represents the more evolutionarily dynamic component of the viral genome, while NA follows a comparatively more conserved and stable evolutionary trajectory.

Overall, the gradual thinning of lineages and the stabilization of demographic patterns suggest ongoing lineage extinction and clade replacement, culminating in the predominance of the contemporary 6B.1A.5a.2a.1 lineage. Taken together, PCA, genetic diversity, BSP, LTT, and selection analyses converge on a coherent picture: compared with the pre-pandemic period, post-COVID-19 A(H1N1)pdm09 evolution in Italy appears to be characterized by reduced genetic diversity, attenuated selective pressure, and a broad genomic restructuring affecting multiple segments. This phase of relative evolutionary stability contrasts with the more turbulent dynamics observed in 2009 and 2023 and suggests that the current viral population may have entered a phase of reduced evolutionary diversification. While such stabilization may facilitate short-term prediction of circulating strains and simplify vaccine strain selection, it does not preclude the possibility of future evolutionary accelerations. Historically, periods of apparent stasis in influenza evolution have often been followed by renewed antigenic diversification, a pattern well documented in studies [66,67,68], showing that increasing population immunity and shifting epidemiological conditions drive episodic bursts of antigenic drift.

These results underscore the importance of maintaining high-resolution genomic surveillance. The integration of phylodynamics, multivariate approaches, genetic diversity and selection analysis provides a powerful framework for detecting early signals of evolutionary reactivation and for anticipating the emergence of novel antigenic variants. Continued monitoring will be essential to understand whether the current evolutionary slowdown represents a transient phase or a more sustained shift in the evolutionary dynamics of A(H1N1)pdm09.

## 5. Conclusions

This study provides an integrated overview of the evolutionary dynamics of A(H1N1)pdm09 viruses circulating in Italy nearly two decades after their emergence. Multivariate, phylodynamic, and genetic diversity analyses consistently indicate that the contemporary viral population is characterized by reduced genetic diversity, attenuated selective pressure, and a marked post-COVID-19 genomic restructuring. The dominance of clade 6B.1A.5a.2a.1, the near absence of positively selected sites, and the lower post-pandemic nucleotide diversity support the view that the virus has entered a phase of relative evolutionary stabilization. Nevertheless, this stabilization does not imply evolutionary stasis. Influenza viruses historically alternate periods of limited diversification with phases of renewed antigenic change as population immunity and epidemiological conditions shift. Strengthening genomic surveillance remains essential to detect early signals of evolutionary reactivation and to anticipate the emergence of novel variants.

It should be noted that genomic data availability may represent a potential source of bias. Sequence availability varied across years and geographical regions, potentially affecting the representation of low-frequency or short-lived lineages. In addition, surveillance data may underrepresent mild or asymptomatic infections, while the present analyses were focused on the HA and NA segments; whole-genome approaches could therefore provide additional insights into viral evolutionary dynamics.

In this context, it is also important to consider that the post-pandemic evolutionary trajectory of A(H1N1)pdm09 is still unfolding. Continued monitoring will therefore be necessary to determine whether the observed stabilization represents a transient phase or a sustained evolutionary pattern.

## Figures and Tables

**Table 1:** Genetic diversity parameters for HA gene segments across pre-pandemic, pandemic, and post-pandemic periods. For each period and gene, the table reports the number of sequences (n), the number of conserved (Cons.) and polymorphic (Poly.) sites, the percentage of polymorphic sites (% Poly.), number of singleton (Singl.) and parsimony-informative (Pars-inf.) sites, number of non-ACGT positions (Non-ACGT), number of haplotypes (Hapl.), haplotype diversity (H.d.), and nucleotide diversity (π).

Gene	Period	n	Cons.	Poly.	% Poly.	Singl.	Pars-inf.	Non-ACGT	Hapl.	H.d.	π
HA	Pre-pandemic	470	1161	540	32	220	320	0	367	0.998	0.021
	Pandemic	111	1483	218	13	63	155	0	72	0.989	0.016
	Post-pandemic	734	1070	631	37	243	388	0	504	0.997	0.017
NA	Pre-pandemic	415	1010	400	28	169	231	2	300	0.993	0.016
	Pandemic	64	1253	157	11	47	110	2	41	0.982	0.014
	Post-pandemic	628	958	452	32	180	272	2	395	0.994	0.009

**Table 2:** Site under positive selective pressure for HA 2025-26 and NA 2025-26. α: Mean posterior synonymous substitution rate at a site; β: mean posterior non-synonymous substitution rate at a site; Prob [α > β]: posterior probability of negative selection at a site; Prob [α < β]: posterior probability of positive selection at a site; BF [α < β]: Empirical Bayes Factor for positive selection at a site; β-α: difference between the non-synonymous and synonymous substitutions indicating the selective pressure on protein-coding sequences.

Gene	Site	Nt Pos.	α	β	β–α	Prob [α > β]	Prob [α < β]	BF [α < β]
HA	468	1402–1404	0.793	5.498	4.705	0.058	0.907	17.032
HA	6	16–18	7.220	0.478	−6.742	0.967	0.022	0.040
HA	9	25–27	3.363	0.516	−2.847	0.910	0.064	0.120
HA	49	145–147	7.408	0.456	−6.952	0.970	0.020	0.037
HA	79	235–237	3.105	0.507	−2.598	0.904	0.069	0.130
HA	91	271–273	5.882	0.452	−5.430	0.962	0.027	0.048
HA	110	328–330	9.433	0.467	−8.967	0.977	0.016	0.028
HA	157	469–471	9.633	0.467	−9.166	0.977	0.016	0.028
HA	165	493–495	4.176	0.516	−3.660	0.928	0.050	0.092
HA	196	586–588	7.853	0.471	−7.382	0.971	0.020	0.036
HA	214	640–642	10.034	0.469	−9.565	0.994	0.003	0.005
HA	239	715–717	9.050	0.486	−8.564	0.974	0.018	0.031
HA	269	805–807	5.894	0.452	−5.442	0.962	0.026	0.048
HA	310	928–930	10.944	0.573	−10.371	0.974	0.016	0.029
HA	362	1084–1086	7.305	0.469	−6.836	0.968	0.021	0.038
HA	366	1096–1098	7.271	0.510	−6.762	0.963	0.024	0.044
HA	329	985–987	3.130	0.511	−2.618	0.905	0.069	0.129
HA	330	988–990	5.979	0.452	−5.526	0.962	0.026	0.047
HA	422	1264–1266	11.056	0.497	−10.559	0.979	0.014	0.025
HA	427	1279–1281	6.290	0.482	−5.807	0.961	0.027	0.048
HA	442	1324–1326	3.079	0.507	−2.572	0.903	0.069	0.131
HA	443	1327–1329	3.107	0.507	−2.600	0.904	0.069	0.129
HA	479	1435–1437	7.234	0.475	−6.759	0.967	0.022	0.040
HA	499	1495–1497	7.777	0.460	−7.317	0.971	0.020	0.035
HA	531	1591–1593	6.884	0.528	−6.356	0.982	0.009	0.016
HA	544	1630–1632	9.311	1.069	−8.241	0.923	0.045	0.082
HA	551	1651–1653	14.523	0.481	−14.042	0.997	0.002	0.003
NA	20	58–60	13.992	0.511	−13.481	0.996	0.002	0.003
NA	69	205–207	5.793	0.493	−5.300	0.952	0.033	0.057
NA	85	253–255	9.021	0.560	−8.460	0.968	0.021	0.035
NA	135	403–405	12.520	0.518	−12.002	0.995	0.002	0.004
NA	205	613–615	9.547	0.499	−9.048	0.976	0.016	0.028
NA	238	712–714	6.585	0.520	−6.066	0.957	0.029	0.051
NA	307	919–921	7.994	0.511	−7.482	0.967	0.022	0.038
NA	353	1057–1059	6.538	0.523	−6.015	0.956	0.030	0.051
NA	400	1198–1200	12.457	0.537	−11.920	0.981	0.012	0.021
NA	401	1201–1203	9.359	0.511	−8.848	0.974	0.017	0.029

## Data Availability

Data used for the analyses are available on GISAID (https://gisaid.org [24], accessed on 5 May 2026). All sequences used were publicly available and free of embargo restrictions at the time of access. Detailed information is provided in Supplementary File S1 (DOI: https://doi.org/10.55876/gis8.260805hc; https://doi.org/10.55876/gis8.260805xg; https://doi.org/10.55876/gis8.260805as; https://doi.org/10.55876/gis8.260805qz).

## Supplementary Materials

The following supporting information can be downloaded at https://www.mdpi.com/article/10.3390/pathogens15090920/s1. Supplementary File S1: Detailed information on the data retrieved from GISAID and used for the analyses.
