# Supplementary material for: Genetic Variability and Evolutionary Dynamics of A(H1N1)pdm09 in Italy Before, During, and After the COVID-19 Pandemic
Source: Pathogens. 2026 Sep 1;15(9):920. doi: 10.3390/pathogens15090920 (PMC13610458; doi:10.3390/pathogens15090920)
Supplement: Supplementary file 1 [file pathogens-15-00920-s001.zip › pathogens-4466769-supplementary.pdf]

Supplementary File S1

All genome sequences and associated metadata supporting the findings of this study can be accessed through the persistent digital object identifier https://doi.org/10.55876/gis8.260805hc

In addition to the minted DOI, GISAID also communicates the aggregation of GISAID accession numbers (EPI_ISL_IDs) through the corresponding EPI_SET_260805hc identifier to facilitate both, the acknowledgment of all data contributors and the direct retrieval of the underlying data from GISAID used in this study.

Influenza Virus Data Summary

GISAID Identifier	Digital Object Identifier	Number of individual viruses	Data Collection range	Number of countries/territories
EPI_SET_260805hc	https://doi.org/10.55876/gis8.260805hc	1,315	2009-05-02 to 2026-02-01	1

Supplementary File S1

All genome sequences and associated metadata supporting the findings of this study can be accessed through the persistent digital object identifier https://doi.org/10.55876/gis8.260805xg

In addition to the minted DOI, GISAID also communicates the aggregation of GISAID accession numbers (EPI_ISL_IDs) through the corresponding EPI_SET_260805xg identifier to facilitate both, the acknowledgment of all data contributors and the direct retrieval of the underlying data from GISAID used in this study.

Influenza Virus Data Summary

GISAID Identifier	Digital Object Identifier	Number of individual viruses	Data Collection range	Number of countries/territories
EPI_SET_260805xg	https://doi.org/10.55876/gis8.260805xg	1,107	2009-05-03 to 2026-02-19	1

Supplementary File S1

All genome sequences and associated metadata supporting the findings of this study can be accessed through the persistent digital object identifier https://doi.org/10.55876/gis8.260805as

In addition to the minted DOI, GISAID also communicates the aggregation of GISAID accession numbers (EPI_ISL_IDs) through the corresponding EPI_SET_260805as identifier to facilitate both, the acknowledgment of all data contributors and the direct retrieval of the underlying data from GISAID used in this study.

Influenza Virus Data Summary

GISAID Identifier	Digital Object Identifier	Number of individual viruses	Data Collection range	Number of countries/territories
EPI_SET_260805as	https://doi.org/10.55876/gis8.260805as	151	2025-10-20 to 2026-02-01	1

Supplementary File S1

All genome sequences and associated metadata supporting the findings of this study can be accessed through the persistent digital object identifier https://doi.org/10.55876/gis8.260805qz

In addition to the minted DOI, GISAID also communicates the aggregation of GISAID accession numbers (EPI_ISL_IDs) through the corresponding EPI_SET_260805qz identifier to facilitate both, the acknowledgment of all data contributors and the direct retrieval of the underlying data from GISAID used in this study.

Influenza Virus Data Summary

GISAID Identifier	Digital Object Identifier	Number of individual viruses	Data Collection range	Number of countries/territories
EPI_SET_260805qz	https://doi.org/10.55876/gis8.260805qz	160	2025-10-20 to 2026-02-19	1
